# A series of methods incorporating deep learning and computer vision techniques in the study of fruit fly (Diptera: Tephritidae) regurgitation

**DOI:** 10.3389/fpls.2023.1337467

**Published:** 2024-01-15

**Authors:** Tongzhou Zhou, Wei Zhan, Mengyuan Xiong

**Affiliations:** ^1^ Department of Computing, The Hong Kong Polytechnic University, Hong Kong, Hong Kong SAR, China; ^2^ School of Computer Science, Yangtze University, Jingzhou, China

**Keywords:** fruit fly, behavior recognition, semantic segmentation, object tracking, regurgitation

## Abstract

In this study, we explored the potential of fruit fly regurgitation as a window to understand complex behaviors, such as predation and defense mechanisms, with implications for species-specific control measures that can enhance fruit quality and yield. We leverage deep learning and computer vision technologies to propose three distinct methodologies that advance the recognition, extraction, and trajectory tracking of fruit fly regurgitation. These methods show promise for broader applications in insect behavioral studies. Our evaluations indicate that the I3D model achieved a Top-1 Accuracy of 96.3% in regurgitation recognition, which is a notable improvement over the C3D and X3D models. The segmentation of the regurgitated substance via a combined U-Net and CBAM framework attains an MIOU of 90.96%, outperforming standard network models. Furthermore, we utilized threshold segmentation and OpenCV for precise quantification of the regurgitation liquid, while the integration of the Yolov5 and DeepSort algorithms provided 99.8% accuracy in fruit fly detection and tracking. The success of these methods suggests their efficacy in fruit fly regurgitation research and their potential as a comprehensive tool for interdisciplinary insect behavior analysis, leading to more efficient and non-destructive insect control strategies in agricultural settings.

## 1 Introduction

In recent years, the harm caused by fruit flies has been aggravated year by year in orchards worldwide and has a great impact on the quality and yield of fruits, even affecting economic growth. Therefore, fruit fly control and the study of their behavior are important.

Since 2017, China’s fruit planting area has gradually increased. The industry has become an important part of China’s agriculture and plays an important role in improving agricultural development and reaching residents’ income. China’s fruit production will reach 300 million tons in 2021, which is 4.5% year-on-year. Among them, apple production is 4,579.34 tons, up 4.3% year-on-year; citrus production is 5,595.61 tons, up 9.2% year-on-year; pear production is 1,887.59 tons, up 6% year-on-year; banana production is 1,172.42 tons, up 1.8% year-on-year. These figures reflect the large production volume of the fruits. However, when growing them, fruits are particularly vulnerable to fruit fly, as Vayssieres et al. (2009) indicated in a 2010 study: fruit fly undermines the quality of mango fruit in Benin, which leads to significant production losses. The maximum loss can exceed 70% and has a significant impact on the economy. According to Badii et al. (2015), fruits and vegetables such as citrus, pineapple, papaya, bananas, and tomatoes are consumed in large amounts every year and create great economic value, but more than 50% of the production is infested by insects. Therefore, many countries and regions are investing heavily in controlling fruit flies, but the results are unsatisfactory, expensive, and inefficient. For example, the use of insecticides for control will be phased out because of the increasingly strict restrictions on the use of insecticides and increasing demand for healthy food worldwide (Dias et al., 2018). Therefore, a green and efficient solution to control fruit flies is urgently needed. Current research on fruit flies is moving in this direction. Ant et al. indicated in a 2012 study, using insect sterility techniques to control fruit fly (Ant et al., 2012). Navarro-Llopis et al. (2014) and Lasa et al. (2013) proposed to apply mass trapping techniques, and trapping devices to trap fruit fly in large scale. The above program is effective in controlling insects and confirms that it is important to study how to control fruit flies from the aspect of physiological habits.

Regurgitation is one of the typical physiological behaviors of fruit fly. Many experts are interesting in it because it contains a variety of behavioral information. Many insects have the behavior as well. For example, herbivorous insects regurgitate at the injury part of plants, and their regurgitation liquid contains inducers that trigger different plant responses. Plants will use the inducers to distinguish mechanical damage and herbivorous insects’ damage so that they can adopt different responses of defense. However, the insects will confuse the plants by creating the wrong kind of inducers so as to suppress plants’ defenses (Timilsena and Mikó, 2017). Regurgitation can also be toxic to vertebrate predators and impose an impact on them (Sword, 2001). Dipteran pests will regurgitate and die without injuring other crops and insects when fed different concentrations of polyols, which provides support for achieving specific insecticide (Díaz-Fleischer et al., 2019). After feeding food the fruit fly, we found that regurgitation can also play an important role in capturing bacteria in the environment and potentially help adult fruit fly to eliminate ingested toxic substances (Guillén et al., 2019). Regurgitation by fly-like insects was also found in a study of Cáceres et al. (2019). According to Wasala et al. (2011), house fly regurgitation spots may be a source of *E. coli* O157:H7 contamination of leafy greens. Therefore, regurgitation liquid can be extracted to detect *E. coli* O157:H7 and other bacteria on plants so that controlling measures can then be taken. In summary, it is important to both study the c behaviors of fruit fly and extract the regurgitated liquids, which will provide practical tools for agricultural experts to do insect’s research.

Current research on regurgitation behavior in fruit flies primarily employs two main methodologies. One involves the detection of regurgitation components using chemical reagents, while the other entails anatomical studies to explore the relationship between regurgitation in fruit flies and their internal structures. It is noteworthy that, as of now, systematic research on the regurgitation behavior of fruit flies using artificial intelligence methods has not been undertaken. With the rapid advancement of deep learning technology and computer vision, these two techniques have found extensive applications in agriculture and insect research, including plant detection, disease identification, and pest control. The introduction of deep learning models facilitates expeditious and efficient conduct of insect and plant research, allowing for tasks such as insect quantification, and the behavioral recognition of various anatomical segments of the citrus fruit fly can be carried out in a straightforward manner. She et al. realized continuous dynamic monitoring of orchards through artificial intelligence technology, helping researchers and fruit farmers grasp disease and insect data in time, reduce the use of artificial and pesticide, and realize scientific early warning and control of diseases and pests (She et al., 2022). Hong et al. (2022) detected the grooming behavior of multi-target insects, providing a research basis for the study of insect-related behaviors.

In this study, we combined deep learning and computer vision techniques and selected three different models for three types of problems in fruit fly regurgitation: behavior recognition, regurgitation liquid extraction, and trajectory tracking. We conducted a comprehensive assessment and comparison of these models to address specific issues, aiming to reveal the underlying mechanisms of regurgitation behavior through an analysis of fruit fly motion trajectories and regurgitation patterns. Our goal was to provide observable and quantifiable data for relevant biological and neuroscientific research. This effort not only contributes to a deeper understanding of fruit fly biological behavior but also establishes a scientific foundation for researchers to delve into the physiological characteristics of fruit flies. Furthermore, we explored the feasibility of applying these methods to other insect regurgitation studies, thereby expanding the potential avenues of research on insect behavior.

## 2 Materials and methods

### 2.1 Overview

The method proposed in this paper to study the regurgitation behavior of fruit flies is divided into three main parts, as follows:

1. Detect and recognize the regurgitation behavior of fruit flies using a behavior recognition network.
2. Use Unet network combined with CBAM attention mechanism and other networks to segment the regurgitated liquid, regurgitated liquid can be extracted precisely, and the area of each liquid can be calculated by OpenCV, so that the total amount of regurgitated liquid can be estimated.
3. To conduct a more comprehensive study of insect regurgitation, we used the Deepsort and Yolov5 method to track the moving trajectory of insects so that their number and trajectory during regurgitation could be recorded simultaneously.

### 2.2 Experimental equipment and environment

The computer equipment used for the behavior recognition experiments is Intel (R) Core (TM) i9-9,900 K CPU @ 3.60 GHz, NVIDIA GeForce RTX 2080Ti with 11G video memory, and the software development environment used was Ubuntu 20.04.1, Python 3.7, and Cuda 11.3. The software development environment used is Ubuntu 20.04.1 operating system, Python environment is 3.7, Cuda 11.3, deep learning framework is PyTorch 1.10.0.

The computer equipment used for the regurgitated liquid extraction and insect trajectory tracking experiments was an 11th Gen Intel (R) Core (TM) i5-11,400 H @ 2.70 GHz 2.69 GHz, and the graphics card was an NVIDIA GeForce RTX 3060 with 6 G of video memory. The Chinese version, python environment is 3.8, Cuda11.5, and deep learning framework is PyTorch 1.10.0.

### 2.3 Model performance metrics

The first part of the behavior recognition experiment was about classification Top-1 Accuracy was used to evaluate the model accuracy. Top-1 Accuracy and Top-5 Accuracy are important metrics used to evaluate the accuracy of the classification model. Top-1 Accuracy refers to tracking the category with the highest probability among the prediction labels as the prediction category, and if the prediction result is the same as the actual result, it is judged to be correct. Top-5 Accuracy refers to taking the top five categories with the highest probability in the prediction labels as the prediction categories, and if one of the categories is the same as the actual result, it is judged to be correct. In the behavior recognition experiment, only fruit fly regurgitation behavior and other behaviors were recognized; therefore, Top-1 Accuracy was chosen as the evaluation index.

**Figure 1:**
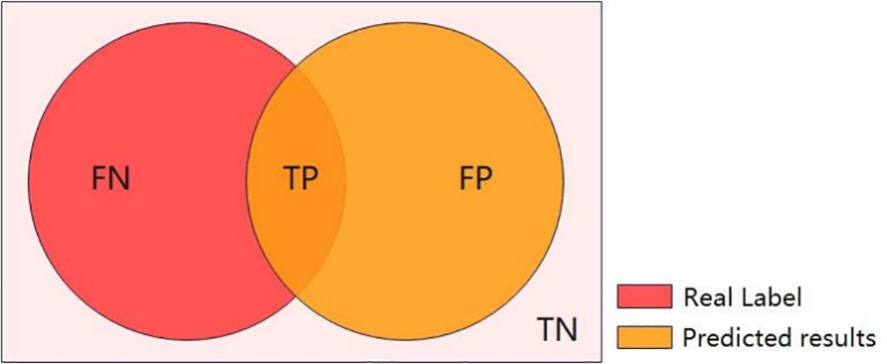
Where true positive (TP) represents that the model is predicted to be a positive case and is actually a positive case. False positive (FP) represents that the model is predicted to be a positive case but is actually a negative case. False negative (FN) represents that the model is predicted to be a negative case but is actually a positive case. True negative (TN) represents that the model is predicted to be a negative case and is actually a negative case.

In the second part of the regurgitation liquid extraction experiment, the main purpose is to evaluate the results of the semantic segmentation experiment and calculate the Miou of semantic segmentation (Miou is Mean Intersection over Union). In semantic segmentation, the intersection and merge ratio of a single category is the ratio of the intersection and merge of the true label to the predicted value of that category (**Figure 1**).

Here the positive cases refer to regurgitated liquids and the negative cases refer to non-regurgitated liquids.

MIoU is the average of the cross-merge ratio for each type of label in this data set. The calculation formula is as follows.


(1) $$MIoU=\frac{1}{k+1}\sum_{i\;=\;0}^{k}\frac{p_{ii}}{\sum_{j\;=\;0}^{k}p_{ij}+\sum_{j\;=\;0}^{k}p_{ji}-p_{ii}}$$

where i denotes the true value, j denotes the predicted value, and p_ij denotes the prediction of i to j. Also equivalent to


(2) $$MIoU=\frac{1}{k+1}\sum_{i\;=\;0}^{k}\frac{TP}{FN+FP+TP}$$

In the third part of the trajectory tracking experiment, two metrics, precision and recall, were used to evaluate the effectiveness of Yolov5 in detecting fruit flies. Precision is a measure of accuracy that describes the number of predicted positive cases that are true positive cases. Here positive cases refer to fruit flies and negative cases refer to non-fruit flies, and are expressed as follows:


(3) $$\begin{array}{c} \text{Precision }=\frac{TP}{TP+FP} \end{array}$$

Recall is a coverage metric that describes how many positive examples are selected from a true outcome perspective, with the following expression.


(4) $$\begin{array}{c} \text{Recall }=\frac{\text{TP}}{\text{TP}+\text{FN}} \end{array}$$

The loss function serves as another evaluation metric for each model training, and is used to estimate the degree of inconsistency between the predicted and true values of the model. This is a non-negative real-valued function. The smaller the loss function, the better is the robustness of the model.

### 2.4 Data collection and processing

During the process of data collection, this study mainly targeted *Bactrocera minax* and *Bactrocera tau*. Both species were photographed at the Insect Ecology Laboratory at the College of Agriculture, Yangtze University. *B. minax* affects almost all fruits of the genus Citrus in the family Rutaceae, and its individuals are relatively large; *B. tau* is smaller in size compared to *B. minax*, and mainly affects squash, cucumber, tomato and other fruits.

To enable the fruit fly to regurgitate, *B. minax* was fed honey water with a concentration of 5% and then placed in closed petri dishes one by one. A video of regurgitation behavior and other actions of the fruit fly was obtained by vertical filming using a Sony video camera (FDR-AX60) with a filming resolution of 1,920 × 1,080 pixels and a filming frame rate of 50 fps.

In the behavioral recognition experiment, the video was edited using several clips. In this study, the video of *B. minax* regurgitation was edited into 50 10 s clips, and that of other actions was edited into 50 10 s clips (other actions include various grooming behaviors and resting states).The 100 video clips collected were divided into training and validation sets at a ratio of 8:2.

In the semantic segmentation experiment, one image was extracted from every 20 frames of the regurgitation video as the dataset for semantic segmentation, and 200 images were obtained. The 200 images of spit water were divided into training and validation sets in 8:2.

In the trajectory tracking experiments, videos containing insects at rest, walking, and while regurgitating were selected, one image was extracted every 50 frames, and 300 images were obtained. The 300 images of the spit-water trajectories were divided into training and validation sets at an 8:2 ratio. This part of the experiment used *B. tau*, which is much smaller than *B. minax*. Because of its small size, it is more difficult to track and test whether the network meets the criteria for tracking insects. Additionally, the Petri dish is limited in size, so smaller-sized fruit flies will more random, which will make the experimental results more precise.

### 2.5 Regurgitation behavior recognition experiment

The behavior recognition task involves identifying different behavioral actions from the video, and the actions can occur continuously or intermittently. Behavior recognition seems to be an extension of the image classification task to multiframe detection, and then aggregating the predictions for each frame. Traditional behavior recognition focuses on feature extraction from videos. It extracts local high-dimensional visual features of video regions, combines them into fixed-size video-level descriptions, and uses classifiers for final prediction. With the development of deep-learning technology, 2D convolutional neural network (2DCNN) has been applied to behavior recognition. 2DCNN is a two-dimensional matrix of input; therefore, the input video is transformed into images, and the sliding window operation can only be performed on one frame of a single channel. This approach cannot consider inter-frame motion information in the time dimension; therefore, the application of 2DCNN in behavior recognition is not satisfactory. However, with a 3D convolutional neural network (3DCNN), behavior recognition can be performed more effectively. 3DCNN has three dimensions: image width, graphic height, and image channel. The convolutional kernel can move in three directions, and the input of one video outputs another video, which retains the input temporal information to better capture the temporal and spatial information in the video (Asadi-Aghbolaghi et al., 2017; Roig Ripoll, 2017; Urabe et al., 2018; Wu et al., 2019; Rastgoo et al., 2020).

In this study, three typical networks were used for the experiments: 3D Convolutional Networks (C3D) (Tran et al., 2015), inflated 3D ConvNet (I3D) (Carreira and Zisserman, 2017) and Expanding Architectures for Efficient Video Recognition (X3D) (Feichtenhofer, 2020). C3D can be regarded as a breakthrough because it is a relatively early proposal to apply the 3DCNN method to behavior recognition. The study proposes the application of 3D convolutional operations to extract spatial and temporal features from video data for behavior recognition. These 3D feature extractors operate in both the spatial and temporal dimensions, thus capturing motion information in the video stream. This structure can generate information channels from adjacent video frames and perform convolution and subsampling in each channel separately to combine the information from all channels to obtain the final features. Compared with 2DCNN, C3D networks are more suitable for learning spatio-temporal features, which can model temporal information by 3D convolution and 3D pooling, whereas 2D convolution can only learn features spatially. The I3D network transforms 2D into 3D, not only to process time repeatedly, which can be obtained by the temporal inflation of all filters and pooling kernels. The main advantage of this method is that the model parameters can be extended to 3D with pretrained 2D images, which solves the problem of not having 3D pretrained parameters. X3D is a relatively new network model with improvements based on previous networks. The previous 3D network mainly expanded the 2D convolutional neural network in the time dimension. However, expanding the time scale is not necessarily the best choice. It is worth expanding on other scales, such as the total frame length of input data, frame rate of input data, size of input frames, and network width and depth. The network eventually outperformed all previous networks in terms of accuracy while requiring only one-fifth of the previous computations and parameters, and it was found that the network could maintain a low number of channels while maintaining high input pixels.

The above networks are for human action behavior datasets in recognition, such as kinetics, UCF101, HMDB-51 and other datasets. For example, kinetics has 400 classes of datasets, each of which comes from a different YouTube video, and the corresponding human action is extracted from the video in a video segment of approximately 10 s.

**Figure 2:**
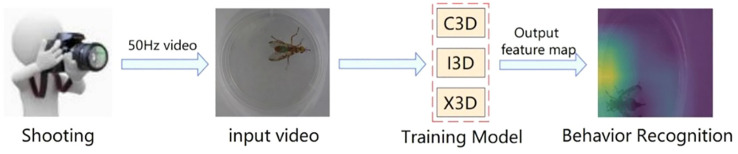
The trained model identifies the insect behavior by the output feature map.

**Figure 3:**
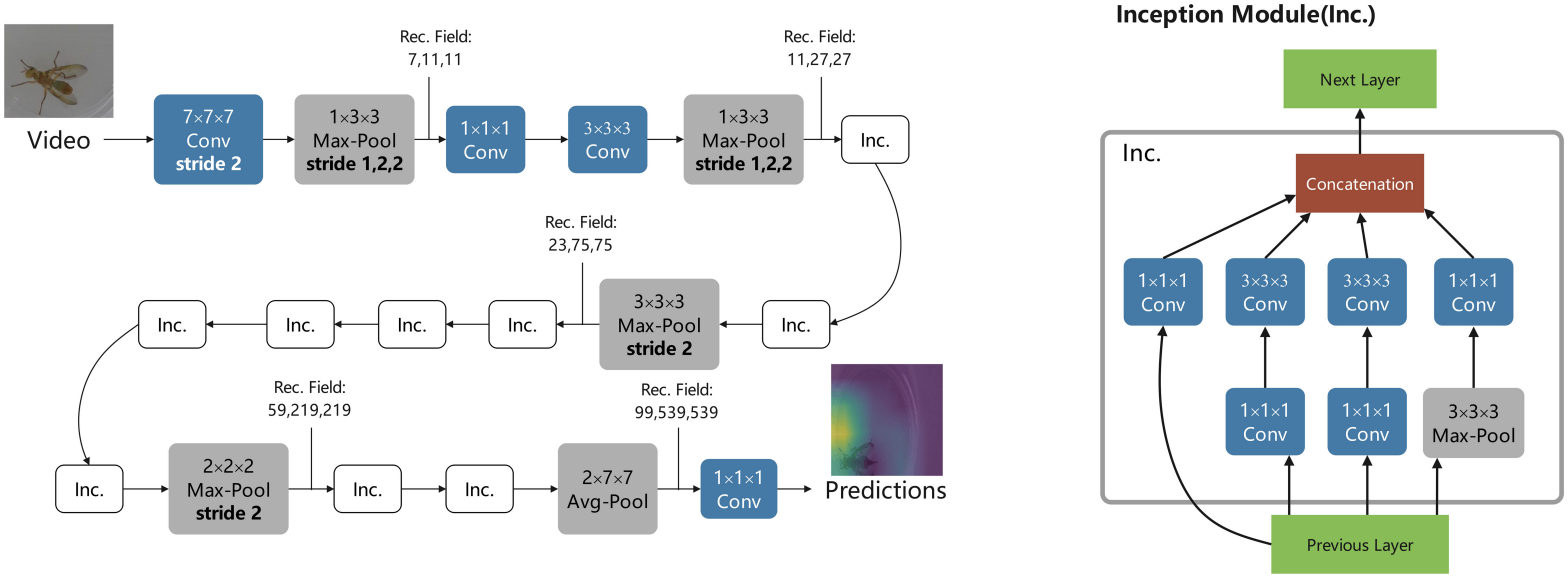
I3D behavior detection model architecture.

The main idea of this study was to expand human behavior recognition to insect behavior recognition. Because insect behaviors are much smaller than human behaviors, we are not sure if the network model can have a good extraction of insect fine action features when detecting their behaviors. Therefore, we confirmed this problem experimentally. We labeled the prepared video clips with data, labeled each small video as an action, and then placed them into C3D, I3D, and X3D networks for training (**Figure 2**). For the recognition of insect spitting behavior, the assigned training sets were placed into C3D, I3D, and X3D networks for iterative training, and the training effects were compared. The model structure of the I3D for water-splitting behavior recognition is shown in **Figure 3**.

### 2.6 Regurgitation liquid extraction experiment

In addition to fruit flies, many insects exhibit regurgitation behavior, which has profound implications for insect ecology and plant interactions. Upon detecting the regurgitation phenomenon in fruit flies during the experiments, we performed semantic segmentation on their regurgitant. Subsequently, threshold segmentation was applied to calculate the area of the regurgitant. Through these research steps, we could accurately measure the area of the droplet, providing crucial data for the quantitative study of fruit fly regurgitation behavior.

In segmenting the regurgitated liquid, the Unet network was firstly used. Why was the Unet network selected? We were inspired by medical image segmentation. Medical semantics are simpler and more fixed in structure. The organ itself is fixed in structure and is not particularly rich in semantic information; therefore, high-level semantic information and low-level features are important. The skip connection and U-shaped structure of Unet combine high-level semantic information and low-level features, making it more suitable for medical semantic segmentation. The fruit fly regurgitated image features are similar to medical images. In other words, the regurgitated liquid resembled a group of ellipse-shaped cells. Its structure is more fixed and the semantic structure is relatively simple; therefore, accurate segmentation is required (Fayemiwo et al., 2021; Zunair and Ben Hamza, 2021; Wang et al., 2022) 

In this study, we evaluated three distinct neural network models, namely C3D, I3D, and X3D, to ascertain their performance and applicability in our domain. As detailed in **Table 1**, each model was configured with identical hyperparameters to ensure a fair and consistent comparison. Specifically, we employed a batch size of 16, a momentum value of 0.9, and utilized the SGD optimizer for training. The initial learning rate was set to 1 X 10-4 across all models. Moreover, each model underwent training for a total of 50 epochs. This uniform setup across the different models allowed us to directly compare their performance and isolate the effects of their unique architectural differences on the task at hand.

**Table 1:** Values of the hyperparameters for the three different network models evaluated in the study.

Model	Batch	Momentum	Optimizer	Initial learning rate	Training epochs
C3D	16	0.9	SGD	1e−4	50
I3D	16	0.9	SGD	1e−4	50
X3D	16	0.9	SGD	1e−4	50

SGD, stochastic gradient descent.

**Figure 4:**
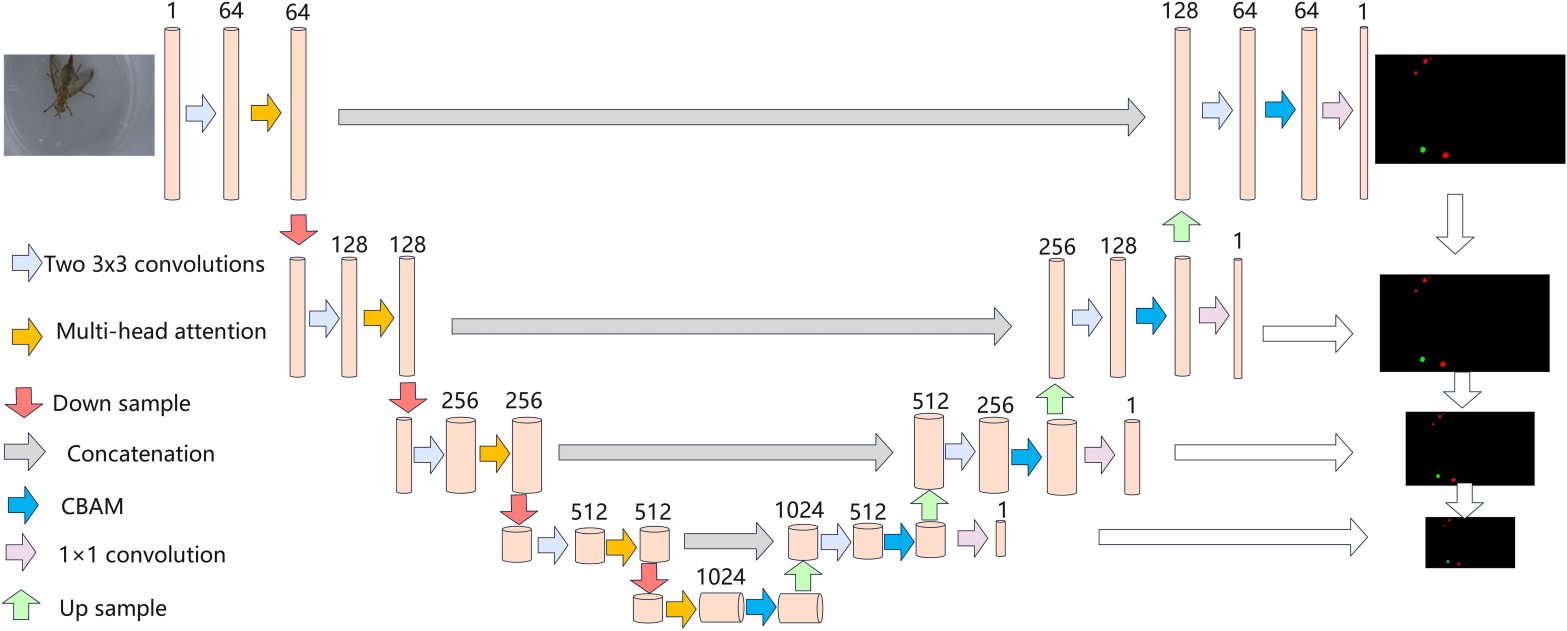
Structure diagram of Unet and CBAM attention mechanism.

To obtain better segmentation results, we attempted to modify the backbone network of Unet using Vgg16 and ResNet50, and then added the CBAM attention mechanism to it, which further improved makes the segmentation effect. The model structure is shown in **Figure 4**. To make the experiments more scientific, ablation experiments are also done in this paper. The semantic segmentation network deeplabv3+ was used, and Xception and MobileNetv2 were also used as the backbone network of DeeplabV3+(Nagrath et al., 2021; Campos et al., 2022; Sutaji and Yildiz, 2022; Xi et al., 2022). The training hyperparameter settings and results are presented in **Table 2**.

**Table 2:** Model performance metrics at different training hyperparameter settings for the two convolutional neural networks evaluated in the study.

Model	Backbone	Optimizer	Initial learning rate	Miou	Loss
Unet	Vgg16	adam	1e−4	89.5	0.056
Unet + CBAM	Vgg16	adam	1e−4	90.96	0.055
Unet	ResNet50	adam	1e−4	85.20	0.096
Unet + CBAM	ResNet50	adam	1e−4	85.95	0.077
deeplabV3+	Xecption	SGD	7e−3	80.69	0.3144
deeplabV3+	MobileNetv2	SGD	7e−3	80.66	0.3211

According to Equation 1 and Equation 2. The mIoU in **Table 2** serves as a key performance indicator for each model, reflecting the average accuracy of the model in segmenting different classes within images. Higher mIoU values signify better segmentation performance, with the Unet + CBAM (Vgg16) model showing the highest mIoU of 90.96%, indicating superior accuracy in class segmentation compared to other models like Unet with ResNet50 or deeplabV3+ with Xception and MobileNetv2. As shown in **Table 2**, Unet’s accuracy is higher when using vgg16 as the backbone, and combining it with the CBAM attention mechanism. Therefore, the training weights of this model were chosen to segment the randomly selected fruit fly regurgitated images. Before segmentation, a square millimeter piece of labeled paper was placed in a petri dish as a “scale” and photographed together with the fruit fly so that the bottom area of the regurgitated liquid could be derived from the pixels of the regurgitated liquid through the area and pixels of the paper.

**Figure 5:**
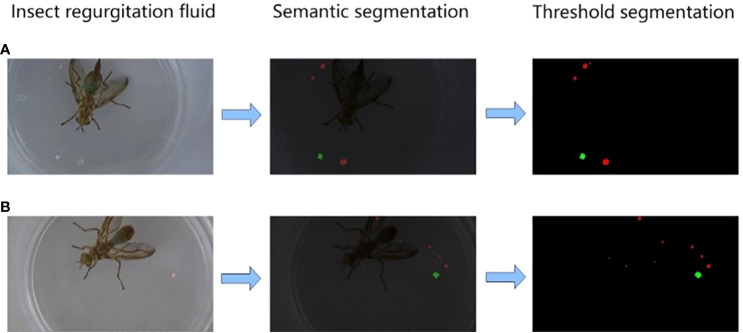
**(A, B)** represent two different sets of samples, and after semantic segmentation and threshold segmentation, a clearer position and shape of the regurgitated liquid can be extracted.

After the segmentation, the extracted regurgitated liquid can be clearly seen, but the segmented image contains impurities, such as the fruit fly themselves and the tiny impurities on the petri dish, which not only bring visual disturbance, but also affect the next step of calculating the regurgitated liquid area. Therefore, the threshold segmentation method can be used to remove impurities and background. Since only regurgitated liquid and marker paper pieces need to be retained, we chose binarization the simplest threshold segmentation, to assign black values to all the impurities and background, and to keep and deepen the color of regurgitated water droplets and marker paper pieces, to obtain a completely extracted regurgitated liquid picture. The extraction process is illustrated in **Figure 5**.

The number of closed shapes in the image and the pixels of each closed shape were calculated using OpenCV, and the area of each liquid was obtained by marking the pixels and area of the paper sheet.

### 2.7 Trajectory tracking experiment

The Yolov5 target detection algorithm combined with the DeepSort algorithm, which has a good tracking performance at present, is used in this study to track the trajectory of fruit flies in the process of regurgitation, and it can realize the counting of fruit flies. We also used the DeepSort network, the most important feature of which is the use of the Kalman filtering algorithm and Hungarian algorithm, both of which can significantly improve the accuracy and speed of multi-object tracking. The Kalman filtering algorithm is divided into two processes: prediction and updating. Prediction: When the target is moved, the target frame position, speed, and other parameters of the current frame are predicted using the target and speed parameters of the previous frame. Update: The two positively distributed states of the predicted and observed values were linearly weighted to obtain the transition state predicted by the current state. In other words, the Kalman filter can predict the position of the current moment based on the position of the target at the previous moment, and can estimate the position of the target more accurately than the sensor. The Hungarian algorithm mainly calculates the similarity to obtain the similarity matrix of the two frames before and after, to determine whether the target in the current frame is the same as the target in the previous frame (Gao and Lee, 2021; Kim and Lee, 2021; Sujin et al., 2021; Ren et al., 2022; Song et al., 2022).

Although the DeepSort network has high accuracy and speed in multi-object tracking, it is mostly used for pedestrian and vehicle tracking and counting. It can achieve good results in tracking objects with relatively large targets and obvious features, but it is seldom used for insect trajectory tracking and counting (Chen et al., 2022; Zhang, 2022). This is because insects are small in size, relatively inconspicuous in features, their trajectories are much smaller than those of straight-line vehicles and pedestrians, and there is no obvious motion pattern. No clear pattern of movement was observed. In this study, we used the DeepSort network to track insects and explored whether there is a network model that can meet the requirements of tracking insects.

**Figure 6:**
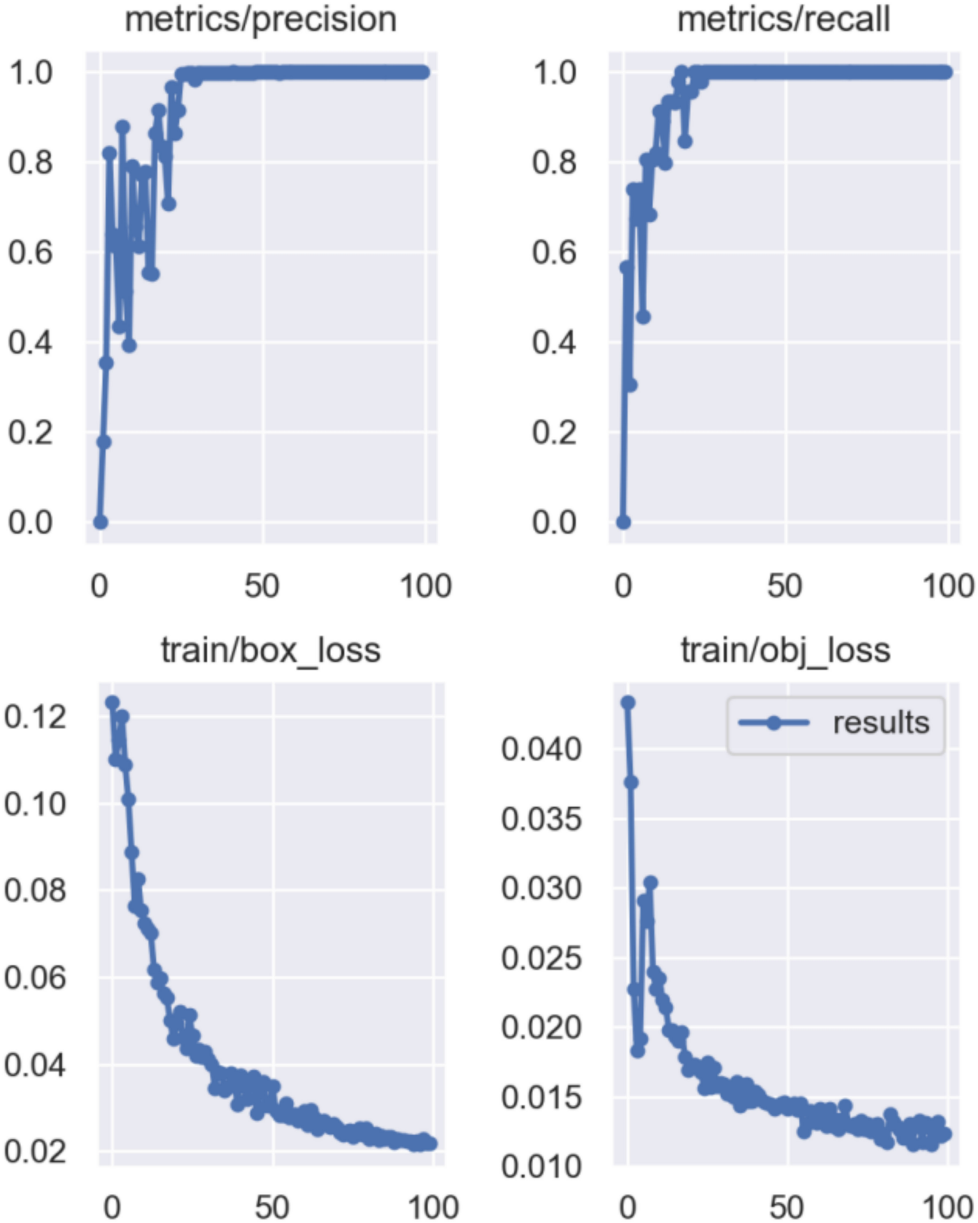
Training accuracy, recall rate and training loss graph of Yolov5 network.

Therefore, 270 images of fruit flies were used to train the Yolov5 network, and 30 images were used to verify its effect. As shown in **Figure 6**, we utilize precision and recall, as defined in Equations 3 and 4, to evaluate the training and testing performance of the YoloV5 model. These metrics effectively reflect the model's accuracy and reliability in object detection tasks. after 50 iterations of training, the accuracy of the network reached 99.8%. The best weights of Yolov5 training were used as the weights of DeepSort object tracking for the tracking experiments. Two South Asian solid flies were detected and tracked using a video.

## 3 Results and discussion

### 3.1 Regurgitation behavior recognition

**Figure 7:**
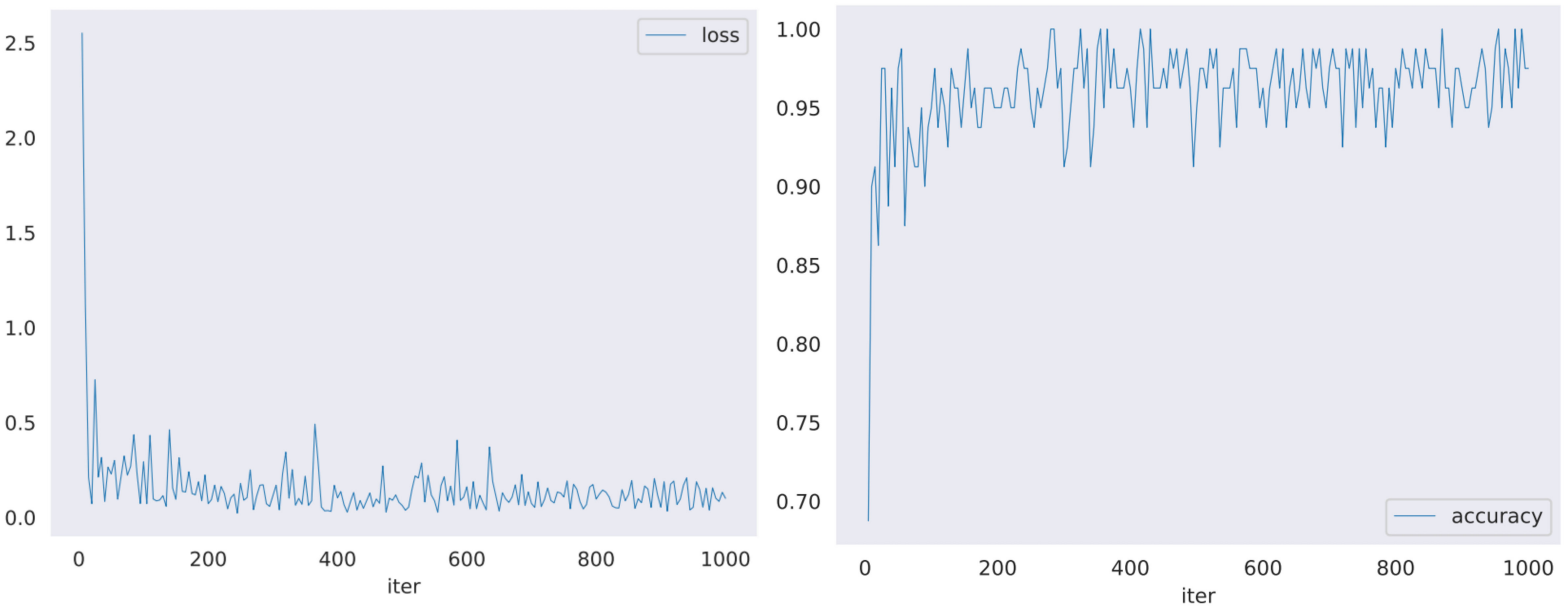
Training accuracy and loss diagram of I3D network.

The same video data and labels were input into the model for training, and the experimental results with the same configuration parameters are listed in **Table 3**. Although the X3D network is more advanced and has better performance in human behavior recognition, it was found through this experiment that after applying it to insect regurgitation behavior recognition, the I3D network performs better with higher accuracy and less training loss, as shown in **Figure 7**. However, from the perspective of training time, the I3D model was still slightly inadequate, and the training time for each item was longer than that of the other models. The training time does not directly affect the detection effect of the behavior recognition experiments; however, if the model is promoted, it will affect the efficiency of the experiment when training many different datasets. Therefore, improving the network model will also become the main focus of later experiments. We planned to replace the network backbone with a lightweight network such as MobileNet, which can improve the training speed while ensuring accuracy (**Table 3**).

**Table 3:** Model performance metrics for the three different network models evaluated in the study.

Network Model	Top-1 Accuracy	Loss	Training times/item
C3D	0.95	0.170	4.67
I3D	0.963	0.101	5.01
X3D	0.925	0.215	4.77

From the results, we find that the idea of transferring human behavior recognition to insect behavior recognition is feasible. All three networks can effectively recognize the regurgitation behavior of fruit flies, and we chose the I3D model as the best performer. This also suggests that we can extend this experiment to behavioral recognition beyond fruit fly regurgitation to recognize all behaviors of fruit flies and similar insects, including forefoot, hindfoot, and head grooming behaviors.

### 3.2 Regurgitation liquid extraction

The regurgitated liquid picture was segmented twice and the liquid area calculated by OpenCV is shown in **Table 4**.

**Table 4:** Number and area of regurgitated liquid beads extracted in **Figures 3A, B**.

Insect regurgitation liquid	Item	Pixel (pt)	Area (mm^2^)	Totalarea (mm^2^)
a	piece of labeled paper	2,455.5	1	1
	Liquid bead No.1	894.5	0.36	1.27
	Liquid bead No.2	165.5	0.06	
	Liquid bead No.3	845.0	0.34	
	Liquid bead No.4	242.5	0.10	
	Liquid bead No.5	62.5	0.03	
	Liquid bead No.6	331.0	0.13	
	Liquid bead No.7	325.0	0.13	
	Liquid bead No.8	293.0	0.12	
b	piece of labeled paper	2,059.5	1	1
	Liquid bead No.1	235.5	0.11	2.64
	Liquid bead No.2	2,759.5	1.34	
	Liquid bead No.3	760.5	0.37	
	Liquid bead No.4	1,698.5	0.82	

This method allows for a general assessment of the amount of regurgitation. Although there are no precise results, this is still beneficial to the analysis of regurgitation research. The experimental results were not completely accurate because the liquid beads were too small, the resolution of the pictures was not high enough, and the liquid beads and background were blurred when the pictures were enlarged and labeled, resulting in less accurate labeling. In addition, the colors of the background and the liquid beads are similar, which makes it difficult to distinguish the liquid beads from the background.

When studying insect regurgitation in a real environment, such as a fruit fly, it is difficult to observe it with the naked eye. The regurgitation behavior occurs for a short period of time and at a high frequency, so it is difficult to know the number of regurgitations even if it is observed with the naked eye. In contrast, the method used in our experiment can clearly mark the number of regurgitated water droplets and extract the approximate area. As mentioned in the introduction, house fly regurgitation spots bring *E. coli* O157:H7 to the vegetables. This problem can be solved by extracting regurgitated liquid. As the study of insects goes deeper, the extraction of insect footprints and the segmentation of individuals are also important, as they can extract different insects from the complex environment and realize the study of insect numbers and individual behavior in many aspects, which is also a prerequisite step for the intelligent diagnosis of pests.

### 3.3 Trajectory tracking

**Figure 8:**
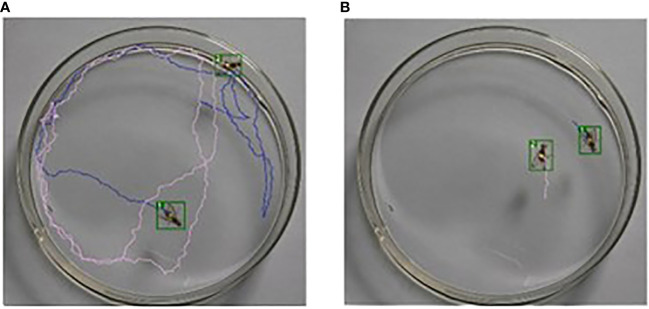
**(A)** the petri dish recording all the trajectories and **(B)** the petri dish recording the first 30 frames of the fruit fly trajectory. The latter avoided the messiness of the trajectory.

To observe the trajectory of the fruit flies more clearly, two recording methods were used. The observer can clearly see the trajectory of the fruit fly by combining the two videos as shown in **Figure 8**.

Tracking the trajectory of fruit flies during their regurgitation can complete the study of fruit fly regurgitation. Additionally, we can apply this model to track and count the trajectories of other insects, which can help analyze insect movement patterns, male/female relationships, and predatory behavior.

## 4 Conclusions

This work demonstrates the feasibility of using deep learning and computer vision techniques to study regurgitation in fruit flies. The proposed method was divided into three main parts. The first is to apply the I3D network to fruit fly regurgitation behavior recognition, the accuracy of which can reach 96.3%. The second step is to segment the extracted regurgitation liquid. The proposed Unet combined with the CBAM attention mechanism model achieves an MIOU of 90.96%, which is 1.46 percentage points higher than the original Unet network and 5–10 percentage points higher than the other network models. Then, threshold segmentation was conducted to obtain the regurgitated liquid quantity and area. The third was to track the trajectory of the fruit fly during its regurgitation by Yolov5+DeepSort. The accuracy of Yolov5 detection of fruit flies was stabilized at 99.8%, and the final tracking effect was satisfactory. The method can be used in fruit fly and other insect regurgitation studies; more importantly, it can be extended to take advantage of deep learning to solve manual observation problems and apply to more insect research tasks according to different needs, which can realize non-destructive research and real-time monitoring of insects.

The current study is only part of fruit fly regurgitation research, and the following three parts will be implemented based on this study. 1. Identification of changes in the mouthparts of fruit flies during regurgitation. When ruminating, the mouthparts of the fruit fly perform specific movements. Although this study was able to detect regurgitation behavior through this feature, there was no detection or visual analysis of the specific movement changes and movement occurrence pattern of the mouthparts. 2. Estimation of the volume of liquid beads regurgitated by fruit fly. In the current research, we extracted the regurgitated liquid using filter paper, which is a traditional method. However, there are errors in this method, and the experimental process is more complicated and time-consuming. An artificial intelligence method to automatically measure the regurgitated liquid volume will be of great help in fruit fly regurgitation research. 3. Realize counting of regurgitated liquid per unit time. This study can realize the counting of regurgitated liquid beads of fruit fly, but our limitation is that it cannot be set flexibly to count the number of regurgitated liquid beads per unit time, which will also be the next focus of work. It is also particularly important to count regurgitated fluid beads in different fruit flies at different times depending on the different experimental studies.

## Data availability statement

The original contributions presented in the study are included in the article/supplementary files. Further inquiries can be directed to the corresponding author.

## Ethics statement

The manuscript presents research on animals that do not require ethical approval for their study.

## Author contributions

TZ: Conceptualization, Data curation, Formal analysis, Methodology, Software, Validation, Writing – original draft, Writing – review & editing. WZ: Funding acquisition, Project administration, Resources, Supervision, Validation, Writing – review & editing. MX: Data curation, Formal analysis, Software, Visualization, Writing – original draft, Writing – review & editing.
